# Necrotizing Pneumonia: A Practical Guide for the Clinician

**DOI:** 10.3390/pathogens13110984

**Published:** 2024-11-10

**Authors:** Esha Madhu Kapania, Rodrigo Cavallazzi

**Affiliations:** Division of Pulmonary, Critical Care, and Sleep Disorders, Department of Medicine, University of Louisville, Louisville, KY 40202, USA; esha.kapania@louisville.edu

**Keywords:** necrotizing pneumonia, sepsis, pulmonary gangrene, septic emboli

## Abstract

While rare, necrotizing pneumonia is a severe and potentially life-threatening manifestation of lung parenchyma infection. Initially documented in the 1940s, it was a significant contributor to mortality rates in both adults and children, with figures reaching up to 45%. Despite being a disease described in the literature for decades, data on the management of necrotizing pneumonia remain limited. Most available information comes from retrospective observational cohort studies. This article aims to provide a comprehensive summary of the existing literature on the subject.

## 1. Introduction

While rare, necrotizing pneumonia is a severe and potentially life-threatening manifestation of a lung parenchyma infection. Initially documented in the 1940s, it stood as a significant contributor to mortality rates in both adults and children, with figures reaching as high as 45% [1,2]. Despite being a disease process that has been described in the literature for decades, there remain very limited data on the management of necrotizing pneumonia. The bulk of the available information derives from retrospective observational cohort studies [3]. This article endeavors to provide a comprehensive summary of the existing literature on this subject.

## 2. Definition

Necrotizing pneumonia arises when a pulmonary infection triggers severe inflammation, leading to parenchymal damage, tissue necrosis, and the liquefaction of consolidated lung tissue [4]. Frequently, the infection results in the destruction of pulmonary vasculature by thrombus formation, thus restricting blood supply to the affected area. This compromised perfusion fosters an environment conducive to lung parenchymal necrosis, uncontrolled bacterial replication, and reduced antibiotic penetration [5]. Consequently, the liquefaction of lung parenchyma can culminate in pulmonary gangrene. The diagnosis of necrotizing pneumonia typically relies on CT imaging findings, which commonly reveal diminished parenchymal contrast enhancement due to inadequate perfusion, alongside the presence of multiple micro-abscesses [4,5]. Although chest X-rays may depict radiolucent lesions suggestive of necrosis, they often lack sensitivity, necessitating CT scans for a definitive diagnosis [5].

Formally diagnosing necrotizing pneumonia presents challenges due to the interchangeable use of terms such as necrotizing pneumonia, lung abscesses, and pulmonary gangrene in the literature. The absence of a unified and standardized definition hampers efforts to study the epidemiology and treatment strategies for necrotizing pneumonia.

Although lung abscesses, necrotizing pneumonia, and pulmonary gangrene share a similar pathological process, distinctions persist in their radiological findings, clinical presentations, and causative pathogens for each of these forms of parenchymal necrosis [3]. A lung abscess typically exhibits a more indolent clinical presentation, with imaging revealing necrosis within a larger cavitary lesion. In contrast, necrotizing pneumonia often manifests with severe clinical illness and sepsis upon presentation. Imaging findings frequently comprise multiple micro-abscesses rather than a single large lesion, along with a more extensive airway involvement than observed in encapsulated lung abscesses. Pulmonary gangrene may be characterized by a greater extent of necrosis (>50% of the affected lobe) and larger vessel thrombosis compared to necrotizing pneumonia [3,6]. It may be more appropriate to consider these three processes as slightly different diseases in the same continuum of necrotizing lung pathology, with pulmonary gangrene being the final stage in the continuum [2,3].

Another distinctive form of infection that causes lung necrosis is septic pulmonary emboli, which is often associated with tricuspid valve infective endocarditis [7]. In this case, lung necrosis is caused by the occlusion of the pulmonary arteries by infected emboli. In most instances, the source of the emboli is the heart, specifically in the form of tricuspid valve infective endocarditis. Peripheral endogenous sources, such as abscesses, phlebitis, and oral infections, or exogenous sources, such as an intravenous line, can also contribute [8]. (See Box 1).

Box 1 **Definitions of lung infections that cause lung necrosis.**

Necrotizing pneumonia: An acute pneumonia that often manifests as sepsis or critical illness and leads to lung parenchymal necrosis. Imaging features include necrosis and micro-abscesses that are often multi-lobar.Lung abscess: A more indolent infection that leads to a large cavitary lesion and is usually isolated to one lobe.Pulmonary gangrene: This is similar to necrotizing pneumonia but with a greater extent of necrosis (>50% of the affected lobe) and larger vessel thrombosis compared to necrotizing pneumoniaSeptic pulmonary emboli: A lung infection characterized by the occlusion of the pulmonary arteries by the infected emboli. The source of the emboli is often tricuspid valve infective endocarditis. Imaging shows bilateral nodular opacities, and cavitation is present in over 50% of the cases.

## 3. Epidemiology

Necrotizing pneumonia is often regarded as a rare manifestation of bacterial lung infections, with prevalence estimates as low as 1% of bacterial pneumonias [5]. Recent studies have raised concerns that the diagnosis of necrotizing pneumonia may be underreported due to missed radiological findings [9]. In a study conducted by Pande et al., re-examination of imaging from a cohort of pneumococcal pneumonia cases revealed a notable increase in the detection of necrotic pneumonia. Initially, evidence of necrotizing pneumonia was found in none of the 351 chest X-rays, but this increased to 8 upon re-evaluation. Similarly, the initial diagnosis of necrotizing pneumonia was found in 6 out of 136 CT scans, but this number increased to 8 upon re-evaluation [9]. Ultimately, the study found evidence of necrotizing changes in 6.6% of the cohort of patients with pneumococcal pneumonia [9]. Another study that re-examined CT scans of patients diagnosed with community-acquired pneumonia (CAP) found evidence of necrotizing changes in 12% of the scans [10]. A similar percentage, 12% of CAP cases being necrotizing pneumonia, is cited in an alternative retrospective study reviewing CT scans of patients hospitalized with CAP [10].

## 4. Etiology

Necrotizing pneumonia is most commonly caused by *Streptococcus pneumoniae*, *Staphylococcus aureus*, and *Klebsiella pneumoniae* [5,11]. This differs slightly from the pediatric population, where *Streptococcus pneumoniae*, *Staphylococcus aureus*, *Mycoplasma pneumoniae*, and *Haemophilus influenzae* are the most common causes [12]. While less common, numerous other organisms have been linked to necrotizing pneumonia in case reports, such as *Escherichia coli, Acinetobacter baumannii*, and occasionally, anaerobes. Most pathogens responsible for lobar pneumonias have been described as being a cause of necrotizing pneumonia. Pseudomonas aeruginosa is rarely associated with necrotizing pneumonia [13]. However, it has been cited as a fatal cause of necrotizing pneumonia in immunocompromised hosts given this pathogen’s ability to invade blood vessels and cause necrosis [11].

## 5. Pathogenesis

As described above, the bacteria common to the etiology of necrotizing pneumonia are also frequently present in pneumonia without necrosis. Micro-aspiration is the primary mechanism by which respiratory pathogens reach the alveoli. The interaction between respiratory pathogens and the airway epithelium leads to activation of Toll-like receptor 2 and the subsequent release of cytokines and chemokines, which stimulate neutrophil migration [14]. Neutrophils are responsible for pathogen phagocytosis [15]. Simultaneously, alveolar macrophages initiate defense against pathogens through pathogen recognition receptors, cytokine and chemokine release, and pathogen phagocytosis [16].

These steps in the pathogenesis are not exclusive to necrotizing pneumonia. A distinguishing feature of necrotizing pneumonia is the role bacterial toxins often play in the pathogenesis. The presence of such exotoxins has been associated with higher mortality rates [4]. Panton–Valentine leucocidin (PVL), an exotoxin found in less than 5% of *Methicillin-resistant Staphylococcus aureus* (MRSA) and *Methicillin-susceptible Staphylococcus aureus* (MSSA) strains, is now thought to play an increasingly important role in the development of necrotizing pneumonia [17]. One case series found that 86% of cases of staphylococcal necrotizing pneumonia were PVL positive [18]. PVL is a pore-forming toxin that, in animal studies, has been found to recruit neutrophils and cause inflammation, resulting in necrosis of lung tissue [19]. One study found that cells exposed to PVL toxin exhibited a disordered release of pro- and anti-inflammatory cytokines, leading to alveolar macrophage dysfunction [20]. Additionally, PVL creates lytic pores in the membranes of neutrophils, promoting the release of chemotactic factors that mediate tissue necrosis [21,22].

**Figure 1:**
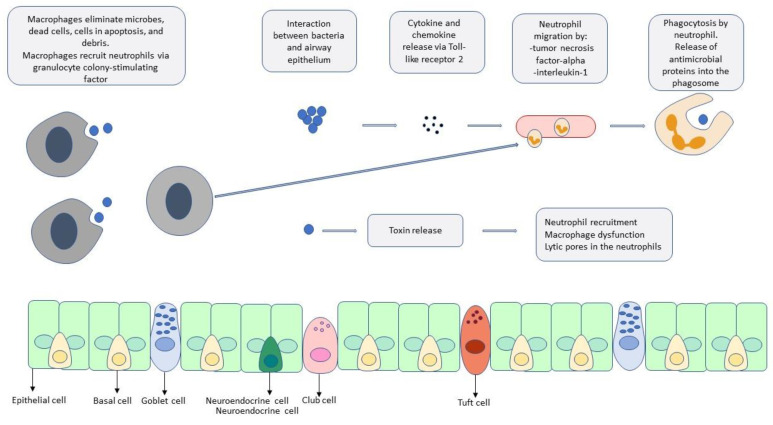
A depiction of the pathogenesis of pneumonia. The interaction of the pathogens with alveolar macrophages and the airway initiates the host response with the local and systemic release of inflammatory mediators and neutrophil recruitment. A distinguishing feature of necrotizing pneumonia is the role that bacterial toxins often play in the pathogenesis.

Similarly, serotype 3 pneumococcal infections, a non-vaccine serotype, have been identified as the most common serotype in cases of necrotizing pneumonia caused by *S. pneumoniae* [9]. One study found that serotype 3 was almost 15 times more likely to be associated with necrotizing pneumonia than other serotypes of pneumococcal pneumonia [23]. The rapid accumulation of capsular polysaccharides associated with serotype 3 is thought to impede the phagocytic activity of alveolar macrophages [11]. Serotype 3 has also been associated with the production of toxins such as leucocidin, hemolysin, and pneumolysin, which have been linked to lung necrosis [24]. See Figure 1 for a depiction of the cellular pathogenesis of pneumonia.

## 6. Risk Factors

In addition to pathogen-related factors contributing to the development of necrotizing pneumonia, numerous clinical risk factors have also been identified. For instance, influenza co-infection has proven to be a major risk factor for developing necrotizing pneumonia. It is speculated that the virus impairs macrophage activity, thereby inhibiting effective bacterial clearance [5]. This risk factor is so well-documented that it has been suggested that a higher degree of suspicion for necrotizing pathology should be considered when treating a bacterial superimposed infection following an influenza-like illness. Similarly, neutropenic patients experience the same impairment in a phagocytic response, predisposing them to the development of necrotizing pneumonia [4]. Unsurprisingly, the presence of leukopenia has been identified as the primary biological feature associated with mortality [5].

Other demographic features, such as smoking, excessive alcohol use, liver disease, and diabetes, have been found to be more prevalent in cases of necrotizing pneumonia compared to non-necrotizing pneumonia [10]. Table 1 highlights the co-morbidities identified in a wide range of cohort studies across various patient populations and hospital centers [2,9,25,26,27,28].

## 7. Clinical Manifestations

Compared to non-necrotizing pneumonia, necrotizing pneumonia is associated with higher rates of complications and more severe clinical manifestations. One study found that 47% of patients with necrotizing pneumonia had complicated parapneumonic effusions or empyema, compared to just 6% in non-necrotizing pneumonia. Additionally, 16% of necrotizing pneumonia patients experienced hemoptysis, whereas only 9% of those with non-necrotizing pneumonia did. These findings align with the higher levels of inflammatory markers observed in necrotizing pneumonia, such as elevated leukocytosis, ESR, and CRP. Unsurprisingly, patients with necrotizing pneumonia also had longer hospital stays, reflecting the increased severity and complexity of their condition [10,25].

Necrotizing pneumonia is clinically characterized by a rapid onset of symptoms, with patients often presenting with signs of sepsis [5], in contrast to lung abscesses, where patients usually experience weeks of fevers, sweats, and a more indolent course. Necrotizing pneumonia is also associated with higher rates of bacteremia and the need for pleural drainage [5]. As a result, complications like ventilation requirements, empyema, and bronchopleural fistulas are common [25]. Hemoptysis, a complication of necrotizing pneumonia, carries a higher mortality risk [17].

## 8. Radiology

**Figure 2:**
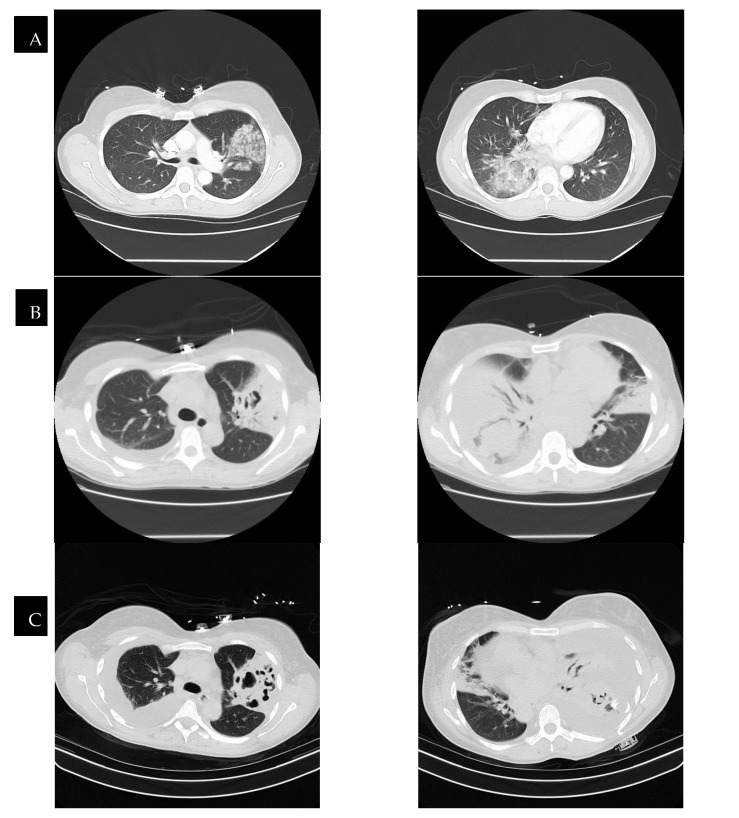

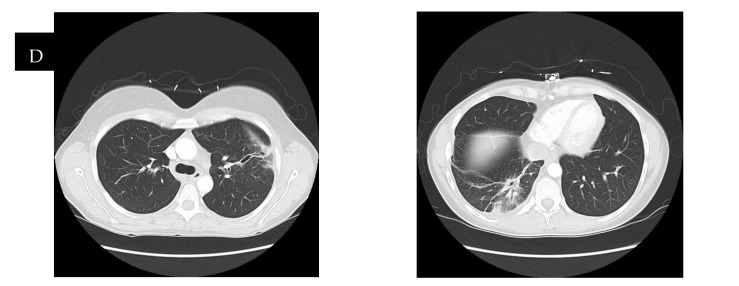
CT scans of a patient with necrotizing pneumonia and progression through time, with row (**D**) representing images after the completion of treatment. The time between images in row (**A**) and row (**D**) is six weeks. Row (**B**,**C**) represent CTs that were taken in between the time of row (**A**) and row (**D**), showing the progression of improvement.

Radiological findings continue to be a mainstay in the diagnosis of necrotizing pneumonia. However, chest X-rays often are not sensitive enough to make the definitive diagnosis of necrotizing pneumonia or to differentiate it from other causes, such as lung abscesses. Bulging fissures may be an early radiographical sign, indicating the extensive inflammation that occurs [3]. A CT with contrast is the optimal imaging modality as it enables the appreciation of areas with low attenuation and decreased enhancement, which are compatible with necrotizing lesions. In contrast to pulmonary abscesses or gangrene, which tend to be isolated to one lobe, necrotizing pneumonia more frequently is multi-lobar in nature and has more extensive airway disease [3,17]. The right middle and lower lobes are most commonly affected in imaging [25]. The presence of micro-abscesses can be used to distinguish necrotizing pneumonia from other conditions causing parenchymal lucency. Significant overlap continues to exist between radiographical findings of necrotizing pneumonia and pulmonary gangrene. Many sources use the extent of lobe involvement as a distinguishing feature, with the term pulmonary gangrene being used when greater than 50% of the lobe is affected [26]. (See Figure 2).

**Figure 3:**
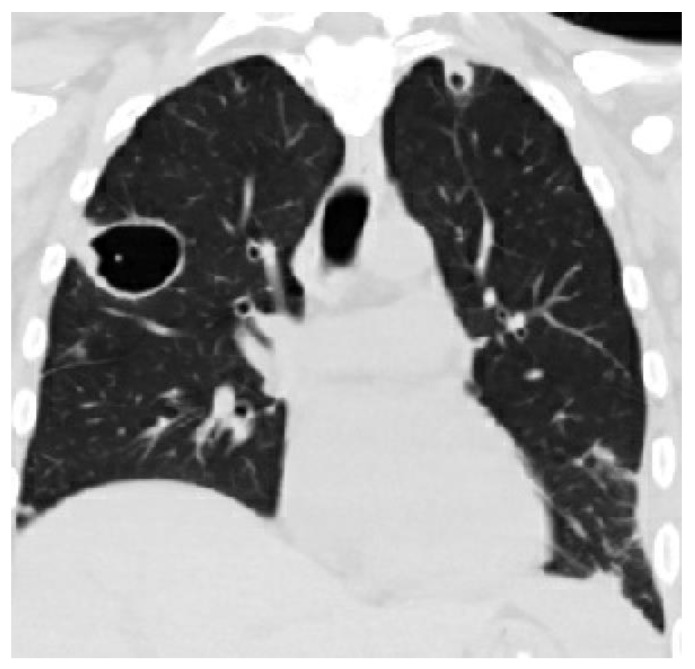
A CT scan of a patient with radiographic evidence of septic emboli.

In patients with septic pulmonary emboli, the chest CT reveals bilateral pulmonary abnormalities in over 80% of the cases. The most common lesions are nodular opacities. Necrosis (cavitation) is seen in slightly over 50% of these patients. Other characteristic but less common lesions include wedge-shaped opacities and the feeding vessel sign (see Figure 3) [7].

## 9. Treatment

Management for necrotizing pneumonia follows most of the same steps as treatment for other types of pneumonia, with the initial step being to isolate the causative organism for tailored antibiotic therapy [5]. Empirical antimicrobial treatment should consider whether the patient acquired the pneumonia in the community or in the hospital [29]. For all patients with necrotizing pneumonia, empirical treatment should cover the core bacterial pathogens causing pneumonia, following IDSA guidelines [29,30]. Our empirical approach has been to select a regimen for treating necrotizing pneumonia according to these guidelines, as outlined in Table 2. Additionally, we recommend a lower threshold for coverage for MRSA when risk factors are present. Indications for including MRSA coverage empirically include prior MRSA colonization or infection, recent hospitalizations, recurrent skin infections [31], and social risk factors such as IV drug use and tobacco use [32]. Additionally, we recommend that, in cases of severe pneumonia and co-infection with influenza, empiric MRSA coverage be implemented [27,31]. In cases of hospital-acquired pneumonia, MRSA coverage should be started empirically if the patient is being treated in units where >10–20% of *S. aureus* isolates are methicillin-resistant, or if the prevalence is not known, in addition to the risk factors outlined above [29]. If MRSA coverage is indicated, Linezolid should be considered over Vancomycin due to its toxin-inhibiting properties, which could benefit patients with necrotizing pneumonia [17]. These patients have an enhanced inflammatory response, especially when co-infected with influenza [27]. Non-lytic antibiotics (e.g., Linezolid), which inhibit bacterial growth and reproduction without lysing the bacteria, thus minimizing the inflammatory response, may be beneficial [17,27]. While this emphasis on non-lytic antibiotics is intriguing, no large, randomized trials confirm their benefit in these patients. While not routinely employed in our empirical antibiotic regimens, clindamycin can be considered as an adjunctive treatment given its toxin-inhibiting properties [17]. We do not believe that empiric oral antibiotic regimes are sufficient in cases of necrotizing pneumonia given the severity of the disease process.

Anaerobes are difficult to culture but are a well-known cause of lung abscesses and empyema. We favor empirical antibiotic regimens that cover anaerobes. In cases of necrotizing pneumonia with concomitant lung abscesses or empyema, anaerobic coverage should be implemented [33]. While Pseudomonas aeruginosa has not been identified as a major cause of necrotizing pneumonias as discussed in the Etiology section above, it is our recommendation to empirically include coverage for *Pseudomonas aeruginosa* when the pneumonia was acquired in the hospital [29].

The duration of antibiotic treatment remains controversial. Recent clinical trials in community-acquired pneumonia suggest benefits from shorter durations of antibiotics in patients who achieve clinical stability [34]. However, shorter therapy durations may not apply to necrotizing pneumonia patients, given the extensive airway and parenchymal involvement. These patients usually receive extended antibiotic courses. Most guidelines on antibiotic duration for necrotizing pneumonia come from the pediatric literature, recommending a 2–4 week course [35]. While biomarkers such as C-reactive protein (CRP) or procalcitonin (PCT) are not recommended for determining whether to start antibiotics for pneumonia [29], there is interesting data supporting the use of biomarkers to guide antibiotic duration. A study by Akagi et al. found that discontinuing antibiotics when procalcitonin levels fell below 0.20 ng/mL resulted in a shorter duration of antibiotic use without increased risk of pneumonia recurrence [36]. However, no studies focus specifically on how biomarkers can guide antibiotic duration in necrotizing pneumonia cases, so caution is advised when using PCT for this purpose. CRP has lower responsiveness than procalcitonin due to its sensitivity to immunosuppressive medications like steroids [37]. Procalcitonin also has a better negative predictive value than CRP [37]. Soluble triggering receptor expressed on myeloid cells (sTREM-1) has been studied as another biomarker that could have clinical utility in pneumonia treatment. However, a meta-analysis conducted as part of the IDSA/ATS guidelines found sTREM-1 to have a sensitivity of 84%, a specificity of 49%, and an AUC of 0.78, indicating moderate accuracy. As such, sTREM-1 is not currently recommended for determining antibiotic therapy in necrotizing pneumonia [29,37].

Adjunctive pharmacological therapies include IVIG and systemic corticosteroids. IVIG has been considered based on animal studies suggesting two specific IVIG antibodies neutralize the toxic effects of Panton–Valentine leucocidin (PVL). However, a multicenter randomized controlled study found no difference in treatment outcomes for severe pneumonia, though the study was not specific to necrotizing pneumonia [38]. We do not routinely prescribe IVIG for patients with necrotizing pneumonia.

Several clinical trials have assessed systemic corticosteroids as immunomodulating agents in patients with community-acquired pneumonia. The rationale for using systemic corticosteroids is to mitigate the enhanced inflammatory response in community-acquired pneumonia. The two most recent clinical trials on this subject had differing outcomes. The CAPE COD trial, which included 795 patients, showed that early hydrocortisone use reduced the 28-day mortality rate in community-acquired infections [39]. Conversely, the ESCAPe trial, involving 584 patients, showed no difference in 60-day mortality for patients treated with methylprednisolone for severe CAP [40]. We have been using systemic corticosteroids in patients with severe pneumonia, including those with necrotizing pneumonia, but we acknowledge the need for further studies in this patient population.

The role and timing of surgery in managing necrotizing pneumonia is a highly debated topic. Numerous case studies have analyzed surgical utility in specific cases. One opinion holds that surgery should be considered when a patient fails medical management, has persistent or significant hemoptysis, or shows evidence of extensive gangrene [5,25]. Surgical interventions range from cavitary debridement to lobectomy but are associated with high mortality [26]. Given the high mortality, many tertiary care centers now prefer aggressive medical management, including significant bronchoscopy use, prompt drainage of pleural fluid collections, and hemoptysis management via embolization, over surgery [2]. One retrospective study found that aggressive medical management lowered mortality rates for most patients [2]. Therefore, we support aggressive medical management before surgical consultation.

**Figure 4:**
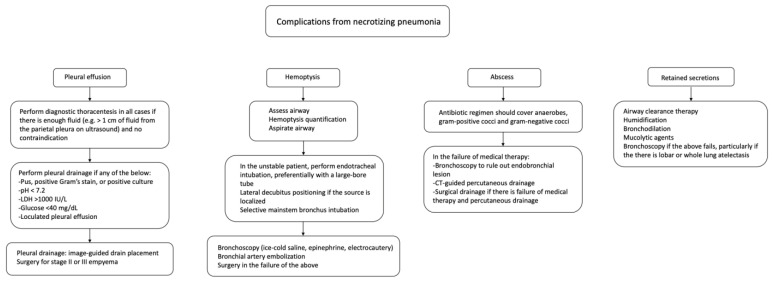
A chart depicting potential complications from necrotizing pneumonia and a summary of the recommendations for management.

We recommend following current guidelines regarding treating complicated parapneumonic effusions in necrotizing pneumonia patients. Specifically, complete drainage via a chest tube is essential for managing these effusions. Additionally, for patients with stage II acute empyema (without an organized pleural peel), surgical consultation for possible Video-Assisted Thoracoscopic Surgery (VATS) should be considered. For mixed stage II/III empyema (areas of fibrinous organization) and stage III (organized pleural peel), surgical consultation for VATS and/or decortication is indicated. Following these guidelines can optimize patient outcomes by managing complications associated with necrotizing pneumonia [41]. See Figure 4 for a summary of necrotizing pneumonia complications and management recommendations [41,42,43,44].

## 10. Prognosis

While there have been significant advancements in imaging, testing, and antibiotic and critical care management of this disease, the overall mortality remains high. The exact mortality rate is difficult to determine given the large variety in how necrotizing pneumonia is defined in the literature. One study found that the overall mortality rate for necrotizing pneumonia was as high as 56% in cases of PVL producing strains of *Staphylococcus aureus* [4]. Many of the same factors associated with an increased rate of mortality in cases of CAP such as the need for mechanical ventilation or pressor support remain true for necrotizing pneumonia as well. In this study, airway bleeding was found to be the clinical manifestation with the highest correlation to death, with leukopenia being the biological feature most associated with death. Erythroderma was also an independent clinical feature associated with fatal outcomes [4].

## 11. Future Directions

Future directions in the field of necrotizing pneumonia necessitate a robust expansion of cohort and prospective studies to better understand the epidemiology, risk factors, and long-term outcomes associated with this severe pulmonary condition. There is a critical need for clinical trials that focus on the optimal duration of antibiotic treatment to balance efficacy and minimize adverse effects. Additionally, research should explore the potential benefits of adjuvant therapies, such as IVIG, for improving patient outcomes. These studies will provide the evidence base required to develop standardized treatment protocols, ultimately enhancing patient care and survival rates in necrotizing pneumonia.

## Figures and Tables

**Table 1:** Epidemiology, co-morbidities, and the mortality of patients with necrotizing pneumonia in cohort studies.

Author, Year	Number of Patients, Clinical Setting	Patient Population	Age/Female Participants	Co-Morbidities	Mortality
Tsai et al. [28], 2011	26 patients, hospital setting	Patients who underwent pulmonary resection for necrotizing pneumonia	64.7 ± 15.0 (mean ± SD) 5 female	Twenty-three (88.5%) patients had underlying risk factors	Four deaths (15.4%) occurred: three due to perioperative progressing pulmonary infection. Postoperative empyema occurred in 3 patients. One patient became ventilator dependent.
Larose et al. [2], 2023	50 patients, single-center hospital setting	All patients hospitalized for severe necrotizing lung infection	55.1 ± 15.8 19 females	Smoker (62%) Alcohol use (24%) IV drug use (5%) Diabetes (22%) CKD (24%) COPD (30%)	4 deaths (8%)
Reimel et al. [26], 2006	35 patients, single-center hospital setting	Patients who underwent parenchymal resection, for necrosis, abscesses, or gangrene	Not available	Not available	3 (8.5%)
Sousa et al. [28], 2013	51 patients, single-center hospital setting	all patients hospitalized with the diagnosis of lung abscess or necrotizing pneumonia	60 9 women	HTN (28%) Smoking (26%) Alcohol use disorder (19%) Pulmonary neoplasm (12%)	9 (18%)
Pande et al. [9], 2012	23 patients, hospital setting	Hospitalized patients for pneumococcal pneumonia	63.6 ± 11.83 years (mean ± SD)	Smoking (78.3%) Alcohol use disorder (47.8%) COPD (39.1%), Heart disease (26.1%), diabetes (13%), malignancy (21.7%), liver disease (26.1%), HIV/AIDS (8.7%)	Not reported

**Table 2:** A summary of antibiotic recommendations for necrotizing pneumonia: community-acquired vs. hospital-acquired approaches.

Pneumonia Type	First-Line Antibiotics and Dosage	When Risk Factors * for MRSA Are Present
Community-acquired pneumonia (CAP)	**Ampicillin-Sulbactam (Unasyn) + Macrolide**: - Ampicillin-Sulbactam: 3 g IV every 6 h - Macrolide (Azithromycin): 500 mg IV on day 1, then 250 mg daily	**Add Linezolid or Vancomycin**: - Linezolid: 600 mg IV every 12 h - Vancomycin: 15–20 mg/kg IV every 8–12 h, adjust for renal function and trough levels.
Hospital-acquired pneumonia (HAP)	**Piperacillin-Tazobactam or Carbapenem**: - Piperacillin-Tazobactam: 4.5 g IV every 6 h, adjust for renal function - Carbapenem (Meropenem): 1 g IV every 8 h	**Add Vancomycin or Linezolid** - Linezolid: 600 mg IV every 12 h - Vancomycin: 15–20 mg/kg IV every 8–12 h, adjust for renal function and trough levels.

* Risk factors for MRSA infection include prior MRSA colonization or infection, recent hospitalizations, recurrent skin infections [31], IV drug use and tobacco use [32], and cases of severe pneumonia and co-infection with influenza [27,31]. In cases of hospital-acquired pneumonia, in addition to the above risk factors, MRSA coverage should be started empirically if patients are being treated in units where >10–20% of *S. aureus* isolates are methicillin-resistant or if the prevalence is not known [29].

## References

[1] Spencer D.A., Thomas M.F. (2014). Necrotising pneumonia in children. Paediatr. Respir. Rev..

[2] Larose J.-C., Wang H.T., Rakovich G. (2023). Survival with optimal medical management in a cohort of severe necrotizing bacterial lung infections. J. Thorac. Dis..

[3] Chatha N., Fortin D., Bosma K.J. (2014). Management of necrotizing pneumonia and pulmonary gangrene: A case series and review of the literature. Can. Respir. J. J. Can. Thorac. Soc..

[4] Gillet Y., Vanhems P., Lina G., Bes M., Vandenesch F., Floret D., Etienne J. (2007). Factors Predicting Mortality in Necrotizing Community-Acquired Pneumonia Caused by Staphylococcus aureus Containing Panton-Valentine Leukocidin. Clin. Infect. Dis..

[5] Krutikov M., Rahman A., Tiberi S. (2019). Necrotizing pneumonia (aetiology, clinical features and management). Curr. Opin. Pulm. Med..

[6] Curry C.A., Fishman E.K., Buckley J.A. (1998). Pulmonary gangrene: Radiological and pathologic correlation. South. Med. J..

[7] Ye R., Zhao L., Wang C., Wu X., Yan H. (2014). Clinical characteristics of septic pulmonary embolism in adults: A systematic review. Respir. Med..

[8] MacMillan J.C., Milstein S.H., Samson P.C. (1978). Clinical spectrum of septic pulmonary embolism and infarction. J. Thorac. Cardiovasc. Surg..

[9] Pande A., Nasir S., Rueda A.M., Matejowsky R., Ramos J., Doshi S., Kulkarni P., Musher D.M. (2012). The Incidence of Necrotizing Changes in Adults With Pneumococcal Pneumonia. Clin. Infect. Dis..

[10] Seo H., Cha S.-I., Shin K.-M., Lim J.-K., Yoo S.-S., Lee J., Lee S.-Y., Kim C.-H., Park J.-Y., Lee W.-K. (2017). Clinical relevance of necrotizing change in patients with community-acquired pneumonia. Respirology.

[11] Tsai Y.-F., Ku Y.-H. (2012). Necrotizing pneumonia: A rare complication of pneumonia requiring special consideration. Curr. Opin. Pulm. Med..

[12] Luo Y., Wang Y. (2023). Clinical Characteristics of Necrotizing Pneumonia Caused by Different Pathogens. Infect. Drug Resist..

[13] Maharaj S., Isache C., Seegobin K., Chang S., Nelson G. (2017). Necrotizing Pseudomonas aeruginosa Community-Acquired Pneumonia: A Case Report and Review of the Literature. Case Rep. Infect. Dis..

[14] Soong G., Reddy B., Sokol S., Adamo R., Prince A. (2004). TLR2 is mobilized into an apical lipid raft receptor complex to signal infection in airway epithelial cells. J. Clin. Investig..

[15] Nauseef W.M. (2007). How human neutrophils kill and degrade microbes: An integrated view. Immunol. Rev..

[16] Quinton L.J., Walkey A.J., Mizgerd J.P. (2018). Integrative Physiology of Pneumonia. Physiol. Rev..

[17] Li H., Zhang T., Huang J., Zhou Y., Zhu J., Wu B. (2010). Factors Associated with the Outcome of Life-Threatening Necrotizing Pneumonia due to Community-Acquired Staphylococcus aureus in Adult and Adolescent Patients. Respiration.

[18] Löffler B., Niemann S., Ehrhardt C., Horn D., Lanckohr C., Lina G., Ludwig S., Peters G. (2013). Pathogenesis of Staphylococcus aureus necrotizing pneumonia: The role of PVL and an influenza coinfection. Expert Rev. Anti Infect. Ther..

[19] Labandeira-Rey M., Couzon F., Boisset S., Brown E.L., Bes M., Benito Y., Barbu E.M., Vazquez V., Höök M., Etienne J. (2007). *Staphylococcus aureus* Panton-Valentine Leukocidin Causes Necrotizing Pneumonia. Science.

[20] Wu B., Zhang W., Huang J., Liu H., Zhang T. (2010). Effect of recombinant Panton-Valentine leukocidin in vitro on apoptosis and cytokine production of human alveolar macrophages. Can. J. Microbiol..

[21] König B., Prévost G., Piémont Y., König W. (1995). Effects of Staphylococcus aureus leukocidins on inflammatory mediator release from human granulocytes. J. Infect. Dis..

[22] Micek S.T., Dunne M., Kollef M.H. (2005). Pleuropulmonary complications of Panton-Valentine leukocidin-positive community-acquired methicillin-resistant Staphylococcus aureus: Importance of treatment with antimicrobials inhibiting exotoxin production. Chest.

[23] Bender J.M., Ampofo K., Korgenski K., Daly J., Pavia A.T., Mason E.O., Byington C.L. (2008). Pneumococcal Necrotizing Pneumonia in Utah: Does Serotype Matter?. Clin. Infect. Dis. Off. Publ. Infect. Dis. Soc. Am..

[24] Hammond J.M.J., Lyddell C., Potgieter P.D., Odell J. (1993). Severe Pneumococcal Pneumonia Complicated by Massive Pulmonary Gangrene. Chest.

[25] Tsai Y.-F., Tsai Y.-T., Ku Y.-H. (2011). Surgical Treatment of 26 Patients with Necrotizing Pneumonia. Eur. Surg. Res..

[26] Reimel B.A., Krishnadasen B., Cuschieri J., Klein M.B., Gross J., Karmy-Jones R. (2006). Surgical management of acute necrotizing lung infections. Can. Respir. J..

[27] McCullers J.A., English B.K. (2008). Improving therapeutic strategies for secondary bacterial pneumonia following influenza. Future Microbiol..

[28] Sousa M., Canelas C., Fontoura I., Rodrigues B., Lemos J., Torres A., Girão F. (2013). Lung abscess and necrotizing pneumonia: A hospital’s experience. Eur. J. Intern. Med..

[29] Kalil A.C., Metersky M.L., Klompas M., Muscedere J., Sweeney D.A., Palmer L.B., Napolitano L.M., O’Grady N.P., Bartlett J.G., Carratalà J. (2016). Management of Adults With Hospital-acquired and Ventilator-associated Pneumonia: 2016 Clinical Practice Guidelines by the Infectious Diseases Society of America and the American Thoracic Society. Clin. Infect. Dis. Off. Publ. Infect. Dis. Soc. Am..

[30] Metlay J.P., Waterer G.W., Long A.C., Anzueto A., Brozek J., Crothers K., Cooley L.A., Dean N.C., Fine M.J., Flanders S.A. (2019). Diagnosis and Treatment of Adults with Community-acquired Pneumonia. An Official Clinical Practice Guideline of the American Thoracic Society and Infectious Diseases Society of America. Am. J. Respir. Crit. Care Med..

[31] Aliberti S., Reyes L.F., Faverio P., Sotgiu G., Dore S., Rodriguez A.H., Soni N.J., Restrepo M.I. (2016). GLIMP investigators Global initiative for meticillin-resistant Staphylococcus aureus pneumonia (GLIMP): An international, observational cohort study. Lancet Infect. Dis..

[32] Lewis P.O. (2021). Risk Factor Evaluation for Methicillin-Resistant Staphylococcus aureus and Pseudomonas aeruginosa in Community-Acquired Pneumonia. Ann. Pharmacother..

[33] Bartlett J.G. (2013). How Important Are Anaerobic Bacteria in Aspiration Pneumonia. Infect. Dis. Clin. N. Am..

[34] Furukawa Y., Luo Y., Funada S., Onishi A., Ostinelli E., Hamza T., Furukawa T.A., Kataoka Y. (2023). Optimal duration of antibiotic treatment for community-acquired pneumonia in adults: A systematic review and duration-effect meta-analysis. BMJ Open.

[35] Bover-Bauza C., Osona B., Gil J.A., Peña-Zarza J.A., Figuerola J. (2021). Long-term outcomes of necrotizing pneumonia. An. Pediatría Engl. Ed..

[36] Akagi T., Nagata N., Wakamatsu K., Harada T., Miyazaki H., Takeda S., Ushijima S., Aoyama T., Yoshida Y., Yatsugi H. (2019). Procalcitonin-Guided Antibiotic Discontinuation Might Shorten the Duration of Antibiotic Treatment Without Increasing Pneumonia Recurrence. Am. J. Med. Sci..

[37] Sungurlu S., Balk R.A. (2018). The Role of Biomarkers in the Diagnosis and Management of Pneumonia. Clin. Chest Med..

[38] Diep B.A., Le V.T.M., Badiou C., Le H.N., Pinheiro M.G., Duong A.H., Wang X., Dip E.C., Aguiar-Alves F., Basuino L. (2016). IVIG-mediated protection against necrotizing pneumonia caused by MRSA. Sci. Transl. Med..

[39] Dequin P.-F., Meziani F., Quenot J.-P., Kamel T., Ricard J.-D., Badie J., Reignier J., Heming N., Plantefève G., Souweine B. (2023). Hydrocortisone in Severe Community-Acquired Pneumonia. N. Engl. J. Med..

[40] Meduri G.U., Shih M.-C., Bridges L., Martin T.J., El-Solh A., Seam N., Davis-Karim A., Umberger R., Anzueto A., Sriram P. (2022). Low-dose methylprednisolone treatment in critically ill patients with severe community-acquired pneumonia. Intensive Care Med..

[41] Shen K.R., Bribriesco A., Crabtree T., Denlinger C., Eby J., Eiken P., Jones D.R., Keshavjee S., Maldonado F., Paul S. (2017). The American Association for Thoracic Surgery consensus guidelines for the management of empyema. J. Thorac. Cardiovasc. Surg..

[42] Karmy-Jones R., Vallières E., Harrington R. (2003). Surgical Management of Necrotizing Pneumonia. Clin. Pulm. Med..

[43] Kathuria H., Hollingsworth H.M., Vilvendhan R., Reardon C. (2020). Management of life-threatening hemoptysis. J. Intensive Care.

[44] New M.L., Huie T.J. (2022). ABCDE Approach for Massive Hemoptysis: A Novel Cognitive Aid. ATS Sch..

